# Association Between eHealth Literacy and Mental Health Literacy: Cross-Sectional Study

**DOI:** 10.2196/76812

**Published:** 2025-12-19

**Authors:** Efrat Neter, Refael Youngmann, Naama Gruper

**Affiliations:** 1 Department of Behavioral Sciences, Ruppin Academic Center, 3 Bait, Emeq Hefer, 4025000, Israel, +972 54-6462677; 2 Clinical Psychology Graduate Program, Ruppin Academic Center, Emeq Hefer, Israel

**Keywords:** eHealth literacy, mental health literacy, stigma, perceived, performance, mental health

## Abstract

Associations between eHealth literacy and mental health literacy were examined; no significant association was identified between overall eHealth and mental health literacy and only weak associations between specific skills were recorded. Results are interpreted in lieu of a difference between perceived ability and actual performance.

## Introduction

As digital technology advances, eHealth literacy (eHL) has become increasingly important for citizens. Defined as the ability to seek, understand, appraise, and apply health information from electronic sources [1], eHL was found to be associated with better health processes [2,3]. Still, it remains unknown whether the skills of using digital resources in health contexts are translated into enhanced mental health (MH) knowledge, such as better identification of MH needs or more accepting attitudes toward seeking support.

Mental health literacy (MHL) refers to knowledge about MH disorders and their recognition, management, and prevention [4]. It encompasses maintaining positive MH, recognizing mental disorders and their treatments, reducing stigma, and enhancing help-seeking efficacy. Low MHL (including stigma) has been linked to the global treatment gap in MH care, persisting across income levels and health care availability [5].

This study thus examined the association between the above two literacies, hypothesizing a positive association, considering potentially overlapping skill sets needed to navigate digital health information and to understand MH.

## Methods

### Participants

The study recruited 269 Hebrew-speaking participants aged ≥18 years; 241 were recruited via an online panel (Panel4All) and 28 using WhatsApp snowball sampling, the latter with no monetary compensation. This study is part of a bigger study assessing MHL in ethnocultural groups in Israel.

### Measures

#### EHL Assessment

eHL was measured using the HLS_19_-DIGI Instrument (Health Literacy Scale 19 - Digital Instrument) [6], comprising 8 items on eHL (Cronbach α=.89). We also analyzed the skills separately: access (3 items, Cronbach α=.69), understand (1 item), appraise (3 items, Cronbach α=.72), and apply (1 item).

#### MHL Assessment

MHL was assessed using items adapted from the Australian National Survey of MHL and Stigma Questionnaire [7], with case vignettes for depression, posttraumatic stress disorder, and schizophrenia, addressing the following dimensions: identification of MH distress, knowledge (potential helpers, appropriate intervention, first aid, all also summed into a general knowledge index), and stigma (9 items, 5-point Likert scale, Cronbach α=0.71‐0.78 for the different vignettes). For more details, see [8].

#### Demographic Characteristics

Information on gender, age, country of birth, ethnicity, education, perceived income adequacy, religiosity, marital status, having children, and self-rated health was collected.

### Ethical Considerations

This cross-sectional survey was conducted online in 2022 following explicit informed consent. The study was approved by the institutional review board of Ruppin Academic Center (approval number 2022‐203) and was registered at OSF (OSF.IO/XMQPC).

### Data Analysis

The characteristics of the participants and the primary variables of the research were described, including bivariate associations (Pearson or Spearman, depending on variable type) between MHL dimensions and eHL.

## Results

### Participants’ Characteristics

Study participants included 151 women (56.1%) and 118 men (43.9%), with a mean age of 42.7 (SD 14.47) years, ranging from 18 to 80 years. They were predominantly Jewish (n=261, 97%) and primarily secular (n=152, 56.7%), the majority had attained higher education (n=154, 57.3%), and financially, they mostly perceived themselves as either living comfortably or managing (n=214, 79.5%). A majority (n=170, 63.1%) rated their health as good or very good.

### Bivariate Associations Between MHL Indices and eHL

Pearson coefficients were computed to examine associations between eHL and MHL indices (Table 1). No significant association was found between the eHL index and indices of MHL (range −0.08 to 0.12), and we therefore computed correlations between items of eHL (assessing, accessing, understanding, applying, and appraising) and MHL indices. A significant positive correlation was found between the perceived skill of *understanding* and knowledge of appropriate interventions and MHL knowledge index (*r*_267_=0.18, *P*=.004 and *r*_267_=0.16, *P*=.01, respectively) and a significant negative correlation between *understanding* and stigma (*r*_267_=−0.15, *P*=.02). Lastly, *applying* was significantly negatively associated with correctly identifying MH conditions. No other association was found between MHL indices and other cognitive aspects of eHL. Controlling for age did not change the results.

**Table 1.** Pearson correlations between mental health literacy indices and digital health literacy skills.

	Access	Understand	Apply	Appraise	Digital health literacy index
Potential helpers	0.04	0.06	0.06	0.02	0.03
Appropriate intervention	0.18	0.18^a^	−0.05	0.00	0.07
First aid	0.11	0.11	0.16^b^	0.05	0.12
MHL^c^ Knowledge Index	0.12	0.16^b^	0.01	0.03	0.09
Stigma	−0.07	−0.15^b^	0.05	−0.06	−0.07
Identification^d^	0.04	0.02	−0.20^a^	−0.08	−0.08

a *P*<.01

b *P*<.05.

c MHL: mental health literacy.

d Spearman correlation in this variable.

## Discussion

We found no significant association between eHL as an index and the different dimensions of MHL, yet we found weak positive associations between the digital skills of understanding and applying and dimensions of knowledge and stigma, while a weak negative association was found between applying and identification of MH conditions.

These findings are consistent with the content of these two measures: the eHL assesses the perceived ease of cognitive activities, whereas the MHL assesses actual performance, that is, the identification of MH conditions and specific knowledge (eg, potential helpers, appropriate interventions), judged in terms of true or false. Indeed, perceptions differ from actual performance, even within the same domain, as evident from modest to nil associations between perceived and performed eHL [9] or in numeracy.

The generalizability of the study is limited by a specific sample, which consisted mostly of highly educated, Jewish, Hebrew-speaking adults with relatively high perceived health and financial stability. Future studies could test the association in broader and more diverse populations and tease apart whether the mostly nil associations are due to measurement (assessing perceptions versus performance) or whether they reflect a genuine lack of association by using different tools.

## Abbreviations:

eHL: electronic health literacy
MH: mental health
MHL: mental health literacy
